# Supplementary material for: Risk of Heart Failure between Different Metabolic States of Health and Weight: A Meta-Analysis of Cohort Studies
Source: Nutrients. 2022 Dec 8;14(24):5223. doi: 10.3390/nu14245223 (PMC9785251; doi:10.3390/nu14245223)
Supplement: Supplementary file 1 [file nutrients-14-05223-s001.zip › nutrients-2053783-supplementary.pdf]

Risk of heart failure between different metabolic states of health and weight: a meta-analysis of cohort studies, Wang et al.

SUPPLEMENTAL MATERIAL

Tables S1–S4

Tables S1. Covariates adjusted in cohort studies.

| Study | Covariates |
|---|---|
| Voulgari, 2011 [14] | Age, sex, fasting glucose, hypertension, lipids, baseline left ventricular hypertrophy and function, current cigarette smoking, and physical inactivity |
| Morkedal, 2014 [15] | Age, sex, smoking status, time since last meal, level of education, marital status, physical activity, and alcohol consumption |
| Caleyachetty, 2017 [18] | Age, sex, self-reported smoking status, and social deprivation |
| Cordola, 2021 [23] | Age, race/ethnicity, income, prevalent diabetes, ever smoking, total healthy eating index score, total energy expenditure from recreational physical activity, and total cholesterol |
| Itoh, 2021 [19] | Age, sex, blood pressure, Glucose, LDL-cholesterol, HDL-cholesterol, triglycerides, cigarette smoking, alcohol drinking |
| Kim, 2021 [20] | Age, sex, income, smoking, alcohol drinking, physical activities, total cholesterol, estimated glomerular filtration rate, medical history and comorbidities |
| Commodore-Mensah, 2021 [30] | age, sex and race-center, smoking status, physical activity, hs-CRP, eGFR, NT-proBNP |
| Zhou, 2021 [16] | age, sex, ethnicity, education, deprivation, smoking, alcohol drinking, television viewing, physical activity, and intake of fruit and vegetables, oily fish, red meat and processed meat |
| Bi, 2021 [31] | sex, education level, smoking status, alcohol drink status, physical activity, LDL-cholesterol and eGFR |
| Fauchier, 2021 [22] | age, sex and smoking status |

Risk of heart failure between different metabolic states of health and weight: a meta-analysis of cohort studies, Wang et al.

Table S2. Subgroup analysis according to sample size*.

| | Small | | | | Large | | | |
|---|---|---|---|---|---|---|---|---|
| | No. of studies | RR (95% CI) | $I^2$ | P for heterogeneity | No. of studies | RR (95% CI) | $I^2$ | P for heterogeneity |
| MHOW | 3 | 1.08 (0.91, 1.21) | 0 | 0.640 | 3 | 1.23 (1.08, 1.39) | 61.1 | 0.08 |
| MHO | 4 | 1.63 (1.40, 1.89) | 21.3 | 0.283 | 3 | 1.49 (1.37, 1.62) | 94.0 | <0.001 |
| MUHNW | 5 | 1.48 (1.24, 1.66) | 15.2 | 0.318 | 2 | 1.35 (1.18, 1.55) | 52.8 | 0.15 |
| MUHOW | 4 | 1.80 (1.52, 2.13) | 59.4 | 0.080 | 2 | 1.83 (1.54, 2.16) | 95.0 | <0.001 |
| MUHO | 5 | 2.19 (1.84, 2.62) | 82.5 | 0.030 | 4 | 2.38 (1.92, 2.95) | 98.8 | <0.001 |

MHOW: Metabolically healthy overweight group, MHO: Metabolically healthy obesity group, MUHNW: Metabolically unhealthy normal-weight group, MUHOW: Metabolically unhealthy overweight group, MUHO: Metabolically unhealthy obesity group.

*sample size less than 100,000 was regarded as small, while over 100,000 was large.

Risk of heart failure between different metabolic states of health and weight: a meta-analysis of cohort studies, Wang et al.

Table S3. Subgroup analysis according to duration of follow-up.

| | <10 years | | | | >10 years | | | |
|---|---|---|---|---|---|---|---|---|
| | No. of studies | RR (95% CI) | $I^2$ | P for heterogeneity | No. of studies | RR (95% CI) | $I^2$ | P for heterogeneity |
| MHOW | 5 | 1.26 (1.22, 1.30) | 8.4 | 0.378 | 2 | 1.03 (0.88, 1.20) | 0 | 0.761 |
| MHO | 4 | 1.63 (1.40, 1.89) | 21.3 | 0.283 | 4 | 1.66 (1.46, 1.89) | 32.1 | 0.219 |
| MUHNW | 5 | 1.89 (1.36, 2.10) | 98.0 | <0.001 | 4 | 1.36 (1.28, 1.45) | 0 | 0.664 |
| MUHOW | 4 | 1.86 (1.82, 2.13) | 90.7 | <0.001 | 2 | 1.50 (1.31, 1.72) | 0 | 0.418 |
| MUHO | 4 | 2.37 (1.89, 2.98) | 98.4 | <0.001 | 4 | 2.19 (1.85, 2.61) | 73.9 | 0.009 |

MHOW: Metabolically healthy overweight group, MHO: Metabolically healthy obesity group, MUHNW: Metabolically unhealthy normal-weight group, MUHOW: Metabolically unhealthy overweight group, MUHO: Metabolically unhealthy obesity group.

Risk of heart failure between different metabolic states of health and weight: a meta-analysis of cohort studies, Wang et al.

Tables S4. Results of sensitivity analyses by omitting one study in each turn and combining the remaining studies.

| Exposure | Omitted study | Combined RR (95% CI) |
|---|---|---|
| Metabolic unhealthy normal weight | | |
| | Voulgari | 1.52 (1.26, 1.84) |
| | Morkedal | 1.60 (1.32, 1.93) |
| | Caleyachetty (Men) | 1.56 (1.27, 1.91) |
| | Caleyachetty (Women) | 1.52 (1.23, 1.87) |
| | Bi | 1.57 (1.29, 1.90) |
| | Commodore-Mensah | 1.59 (1.31, 1.92) |
| | Cordola | 1.55 (1.28, 1.87) |
| | Kim | 1.60 (1.34, 1.91) |
| | Zhou | 1.59 (1.31, 1.91) |
| | Fauchier | 1.48 (1.32, 1.67) |
| Metabolic unhealthy overweight | | |
| | Voulgari | 1.71 (1.51, 1.94) |
| | Morkedal | 1.81 (1.59, 2.05) |
| | Caleyachetty (Men) | 1.77 (1.50, 2.08) |
| | Caleyachetty (Women) | 1.66 (1.55, 1.79) |
| | Bi | 1.76 (1.54, 2.02) |
| | Commodore-Mensah | 1.79 (1.56, 2.04) |
| | Kim | 1.78 (1.54, 2.06) |
| Metabolic unhealthy obesity | | |
| | Voulgari | 2.29 (1.96, 2.68) |
| | Morkedal | 2.36 (2.02, 2.77) |
| | Caleyachetty (Men) | 2.40 (2.16, 2.68) |
| | Caleyachetty (Women) | 2.26 (1.89, 2.70) |

| | | |
|---|---|---|
| | Bi | 2.24 (1.90, 2.64) |
| | Commodore-Mensah | 2.26 (1.92, 2.66) |
| | Cordola | 2.31 (1.97, 2.71) |
| | Zhou | 2.25 (1.87, 2.70) |
| | Fauchier | 2.21 (1.95, 2.51) |
| Metabolic healthy overweight | | |
| | Voulgari | 1.23 (1.17, 1.29) |
| | Morkedal | 1.25 (1.20, 1.30) |
| | Caleyachetty (Men) | 1.20 (1.11, 1.29) |
| | Caleyachetty (Women) | 1.20 (1.12, 1.29) |
| | Bi | 1.24 (1.18, 1.30) |
| | Commodore-Mensah | 1.25 (1.19, 1.30) |
| | Itoh | 1.24 (1.18, 1.30) |
| | Kim | 1.22 (1.16, 1.28) |
| Metabolic healthy obesity | | |
| | Voulgari | 1.52 (1.41, 1.65) |
| | Morkedal | 1.51 (1.39, 1.63) |
| | Caleyachetty (Men) | 1.53 (1.38, 1.70) |
| | Caleyachetty (Women) | 1.53 (1.38, 1.68) |
| | Bi | 1.49 (1.38, 1.62) |
| | Commodore-Mensah | 1.51 (1.39, 1.63) |
| | Cordola | 1.53 (1.41, 1.66) |
| | Zhou | 1.46 (1.36, 1.57) |
| | Fauchier | 1.56 (1.44, 1.69) |

RR: relative risk
